# An Atypical Case of Vanished Gallbladder

**DOI:** 10.7759/cureus.113018

**Published:** 2026-07-20

**Authors:** Ritu Nehra, Mohit K Badgurjar, Sanjeev Agarwal, Yogesh K Baberwal, Dhawal Vyas

**Affiliations:** 1 Department of General Surgery, Geetanjali Medical College and Hospital, Udaipur, IND

**Keywords:** choledocholithiasis, endoscopic retrograde cholangiopancreatography (ercp), gallbladder agenesis, laparoscopic exploration, magnetic resonance cholangiopancreatography (mrcp), vanished gallbladder

## Abstract

Gallbladder agenesis (GA) is an uncommon congenital anomaly that often mimics common biliary disorders, leading to frequent misdiagnosis and unnecessary surgical intervention. We report a case of a 69-year-old woman presenting with biliary symptoms and choledocholithiasis, in whom repeated imaging suggested a contracted gallbladder. GA was ultimately diagnosed intraoperatively during a planned laparoscopic cholecystectomy. This case highlights the diagnostic limitations of conventional imaging, the importance of maintaining suspicion when the gallbladder is persistently non-visualized, and the need for surgical preparedness to avoid biliary injury and improve outcomes.

## Introduction

Gallbladder agenesis (GA) is an uncommon congenital anomaly characterized by complete absence of the gallbladder, with an incidence of approximately 10-65 per 100,000 individuals [1]. Despite its rarity, GA frequently presents with symptoms indistinguishable from common biliary disorders, including right upper quadrant (RUQ) pain, dyspepsia, and biliary obstruction, leading to a high risk of misdiagnosis. Conventional imaging modalities, particularly ultrasonography, often misinterpret gallbladder non-visualization as a contracted or fibrotic gallbladder rather than true agenesis [2].

This diagnostic limitation frequently results in an erroneous preoperative diagnosis of cholecystitis or cholelithiasis, prompting unnecessary surgical intervention, with GA often identified only during laparoscopy. Such unexpected intraoperative findings increase operative complexity and the risk of iatrogenic biliary injury. Additionally, patients with GA may have associated biliary complications, such as choledocholithiasis, further compounding diagnostic and therapeutic challenges [3].

This case report aims to highlight an atypical presentation of a "vanished" gallbladder, emphasizing the diagnostic complexities and the critical need for a high index of suspicion and advanced imaging in the preoperative workup to prevent avoidable surgical complications and improve patient outcomes.

## Case presentation

**Figure 1:**
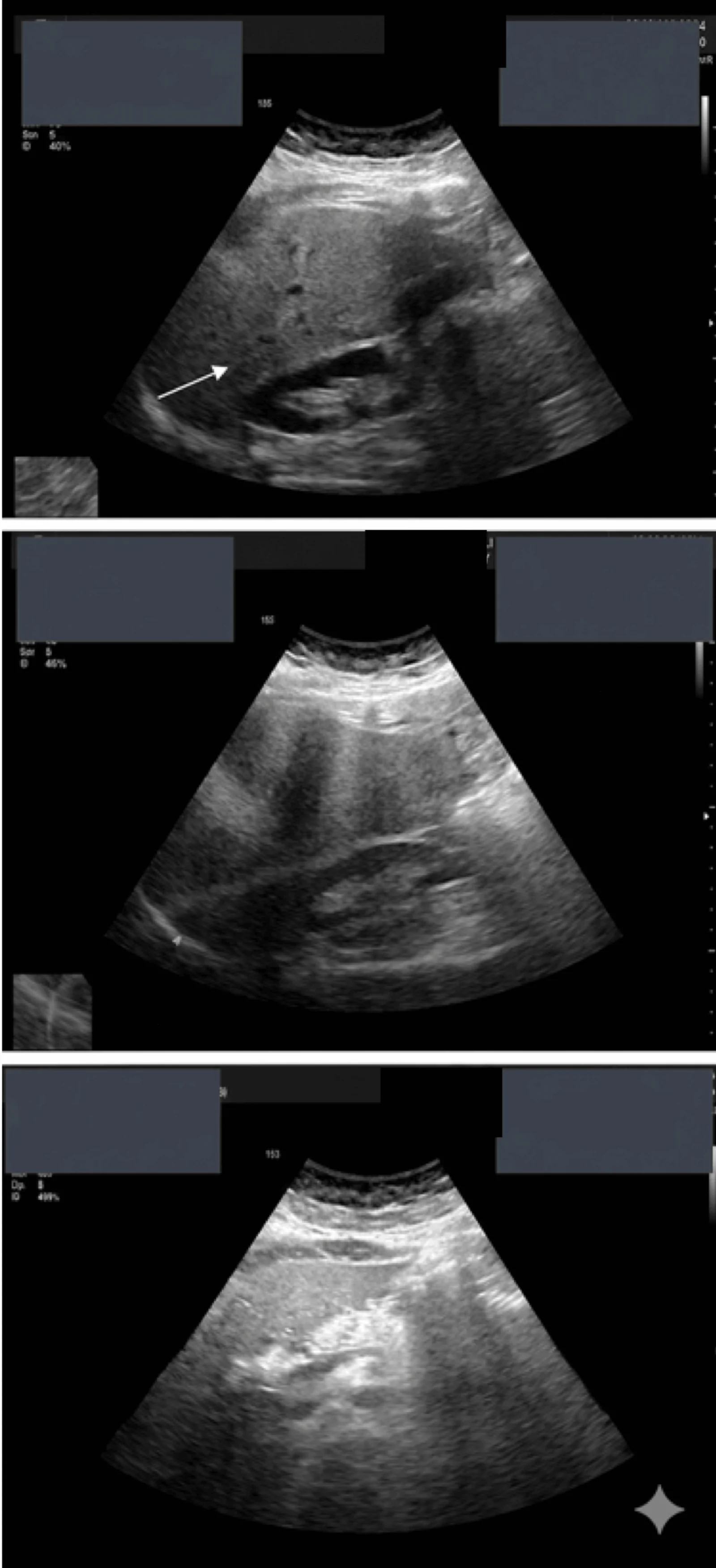
Ultrasonographic images of a 69-year-old woman presenting with chronic right upper quadrant pain and postprandial fullness. Imaging demonstrates a poorly visualized/contracted gallbladder with features suggestive of choledocholithiasis and associated intrahepatic biliary radicle dilatation (IHBRD).

**Figure 2:**
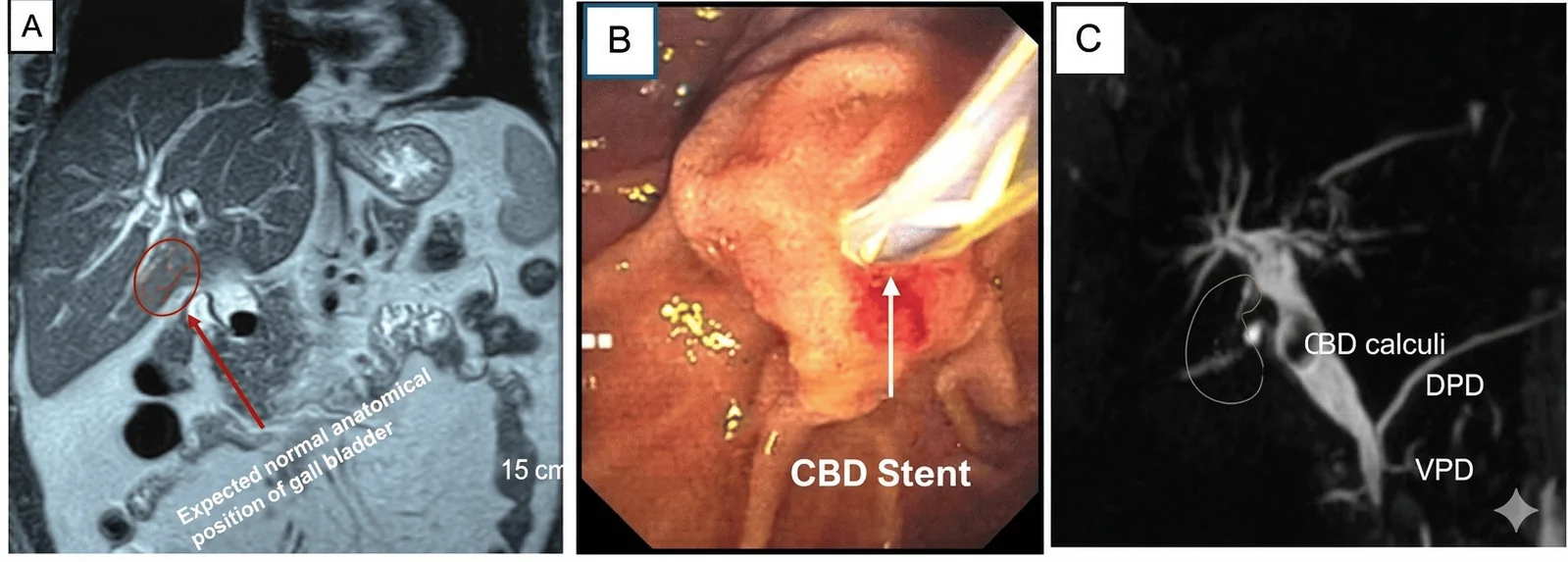
(A) Initial magnetic resonance cholangiopancreatography (MRCP), (B) placement of CBD stent, and (C) repeat MRCP. CBD: common bile duct; DPD: dorsal pancreatic duct; VPD: ventral pancreatic duct.

A 69-year-old woman with known comorbidities of diabetes mellitus, hypertension, and hypothyroidism presented with postprandial fullness and recurrent pain in the RUQ of the abdomen occurring intermittently over one year. There were no features suggestive of acute abdominal pathology. Initial evaluation with ultrasonography demonstrated poor visualization of the gallbladder (Figure 1), suspected to be contracted, along with features of choledocholithiasis and intrahepatic biliary radicle dilatation (IHBRD); correlation with magnetic resonance cholangiopancreatography (MRCP) was advised. MRCP demonstrated a contracted gallbladder (Figure 2A). An oval hypointense filling defect measuring approximately 13 mm was noted within the common bile duct (CBD), causing distal obstruction with proximal CBD dilatation (approximately 15 mm) and moderate IHBRD. The common hepatic duct appeared normal. These findings were suggestive of choledocholithiasis. At presentation, laboratory investigations revealed Hb: 11.4 g/dL, total leukocyte count (TLC): 5,990/mm³, platelet count: 2.87 × 10⁵/mm³, total bilirubin: 1.72 mg/dL, direct bilirubin: 1.1 mg/dL, indirect bilirubin: 0.62 mg/dL, serum glutamic-oxaloacetic transaminase (SGOT): 171 IU/L, serum glutamic-pyruvic transaminase (SGPT): 290 IU/L, and alkaline phosphatase (ALP): 639 IU/L (Tables 1-5).

**Table 1:** Blood investigations. MCH: mean corpuscular hemoglobin; MCHC: mean corpuscular hemoglobin concentration; MCV: mean corpuscular volume; RDW: red cell distribution width; TLC: total leukocyte count; INR: International Normalized Ratio.

Test	Value	Unit	Normal range	Remarks
Hemoglobin (Hb)	11.4	g/dL	12-16 (F), 13-17 (M)	Mildly low
Hematocrit (HCT)	34.9	%	36-46 (F), 40-52 (M)	Mildly low
RBC count	4.36	million/cumm	4.0-5.5 (F), 4.5-6.0 (M)	Normal
MCV	80.1	fL	80-100	Normal
MCH	26.2	pg	27-32	Normal
MCHC	32.7	g/dL	32-36	Normal
RDW	15.4	%	11.5-14.5	Slightly high
TLC	5990	/cumm	4000-11000	Normal
Neutrophils	62	%	40-70	Normal
Lymphocytes	24	%	20-40	Normal
Eosinophils	2	%	1-6	Normal
Monocytes	12	%	2-10	Slightly high
Basophils	0	%	0-1	Normal
Platelet count	2.87	lakhs/cumm	1.5-4.5	Normal
Prothrombin time (PT)	11.2	sec	10-13	Normal
INR	1	-	0.8-1.2	Normal

**Table 2:** Liver parameters. ALT: alanine aminotransferase; AST: aspartate aminotransferase; SGOT: serum glutamic-oxaloacetic transaminase; SGPT: serum glutamic-pyruvic transaminase.

Test	Value	Unit	Normal range	Remarks
Total bilirubin	1.72	mg/dL	0.3-1.2	Elevated
Direct bilirubin	1.1	mg/dL	0.0-0.3	Elevated
Indirect bilirubin	0.62	mg/dL	0.2-0.8	Normal
SGOT (AST)	171	U/L	5-40	Elevated
SGPT (ALT)	200	U/L	5-45	Elevated
Alkaline phosphatase	639	U/L	44-147	Elevated
Total protein	7.34	g/dL	6.0-8.0	Normal
Albumin	3.82	g/dL	3.5-5.0	Normal
Globulin	3.52	g/dL	2.0-3.5	Normal
A/G ratio	1.09	-	1.0-2.0	Normal

**Table 3:** Urine analysis. HPF: high-power field; LPF: low-power field.

Test	Value	Unit	Normal range	Remarks
Colour	Pale yellow	-	Pale yellow	Normal
Appearance	Clear	-	Clear	Normal
pH	6	-	4.5-8.0	Normal
Specific gravity	1.01	-	1.005-1.030	Normal
Urobilinogen	Negative	-	Negative	Normal
Protein	Negative	-	Negative	Normal
Glucose	Negative	-	Negative	Normal
Bile pigment	Negative	-	Negative	Normal
Ketone	Negative	-	Negative	Normal
Blood	Negative	-	Negative	Normal
Nitrite	Negative	-	Negative	Normal
RBC	NIL	/HPF	0-2/HPF	Normal
Pus cells	4-6	/HPF	0-5/HPF	Mildly increased
Epithelial cells	2-3	/HPF	0-5/HPF	Normal
Casts	NIL	/LPF	NIL	Normal
Crystals	NIL	-	NIL	Normal
Bacteria	+	-	Absent	Suggestive of infection
Yeast cells	NIL	-	NIL	Normal

**Table 4:** Pancreatic enzymes and serology. HCV: hepatitis C virus; HBsAg: hepatitis B surface antigen.

Test	Value	Unit	Normal range	Remarks
Serum amylase	38.8	U/L	30-110	Normal
Serum lipase	59.3	U/L	10-60	Normal (upper normal)
HCV	Non-reactive	-	Non-reactive	Negative
HIV 1 & 2	Non-reactive	-	Non-reactive	Negative
HBsAg	Non-reactive	-	Non-reactive	Negative
Anti-HCV	Negative	-	Negative	Negative

**Table 5:** Cardiac (echo) parameters. EF: ejection fraction; RWMA: regional wall motion abnormality; LV: left ventricle; LVH: left ventricular hypertrophy.

Test	Value	Normal/reference	Remarks
LVH	Present	Absent	Concentric LVH
RWMA	Absent	Absent	Normal
Mitral regurgitation (MR)	Mild	None/trace	Mild MR
LV function	Normal	EF: 55-70%	Good function
Diastolic function	Type I	Normal relaxation pattern	Grade I diastolic dysfunction
Clot/vegetation	Absent	Absent	Normal

Subsequently, endoscopic retrograde cholangiopancreatography (ERCP) was performed for biliary obstruction (Figure 2B). Selective cannulation of the CBD was achieved. Cholangiography revealed a massively dilated CBD with a large filling defect measuring approximately 20 mm and distal narrowing. Sphincterotomy and balloon sweep were performed; however, the calculus could not be retrieved. A CBD stent was therefore placed for biliary drainage.

As CBD calculus could not be retrieved during ERCP, repeat MRCP was done, which confirmed a suprapancreatic CBD stone with persistent non-visualization of the gallbladder, again described as likely contracted (Figure 2C).

**Figure 3:**
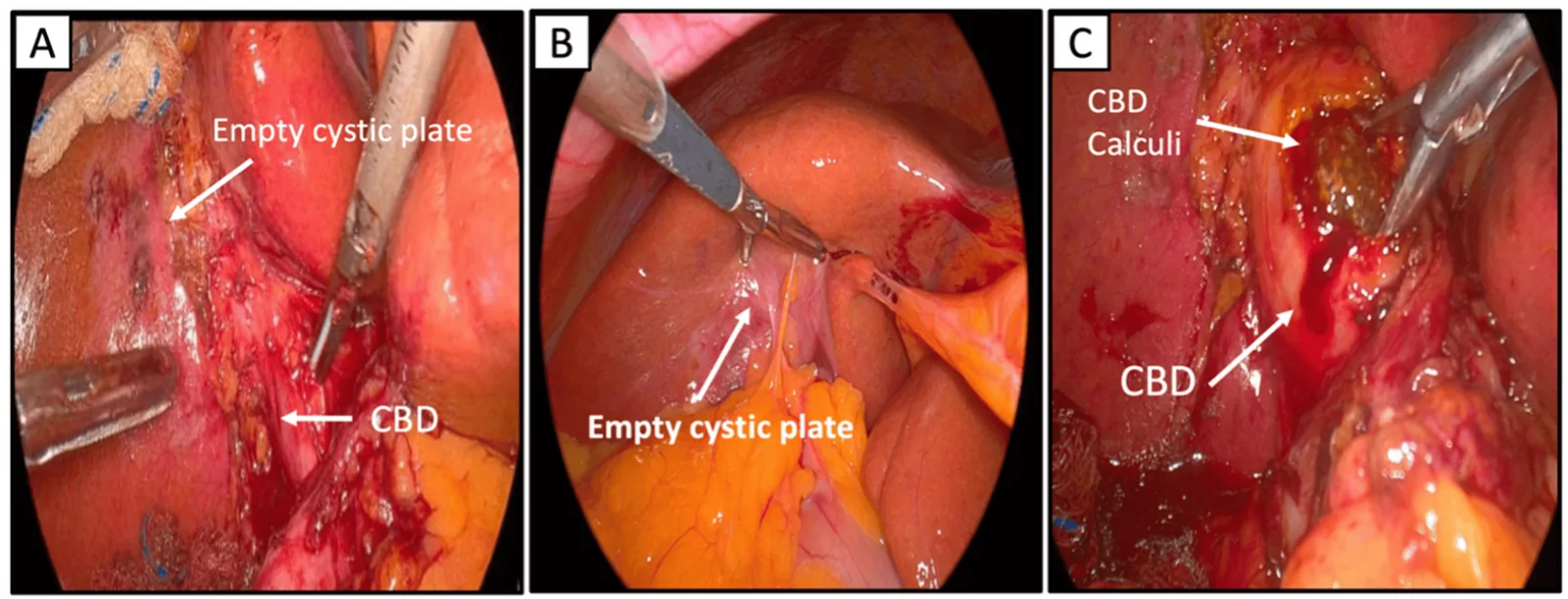
(A)-(C) Laparoscopic cholecystectomy with CBD exploration. CBD: common bile duct.

In view of persistent biliary pathology, laparoscopic cholecystectomy with CBD exploration was planned. Intraoperatively, the gallbladder was found to be absent, consistent with a vanished gallbladder, and the CBD was dilated (Figures 3A-3C).

Laparoscopic CBD exploration was performed, and the CBD stone was successfully removed. The postoperative course was uneventful. The patient was discharged on postoperative day two. Follow-up at seven days revealed no new complaints, and suture removal was performed. Histopathological examination was not done as the gallbladder was absent. Follow-up ultrasonography at one month showed non-visualization of the gallbladder, with mild CBD prominence, minimal central IHBRD, and the biliary stent in situ. ERCP with stent removal was performed the following day.

## Discussion

GA is a rare congenital biliary anomaly, with a reported prevalence of 10-65 per 100,000 individuals and a well-documented female predominance [4]. Symptomatic patients commonly present with postprandial fullness, RUQ pain, nausea, or biliary colic, closely resembling chronic cholecystitis or choledocholithiasis, which contributes substantially to diagnostic error [5]. In the present case, the clinical picture and radiological findings strongly favored biliary pathology, masking the underlying anomaly.

A major diagnostic challenge in this case was the persistent non-visualization of the gallbladder on both ultrasound (US) and MRCP, repeatedly interpreted as a contracted gallbladder. The unusual feature was the persistent radiological interpretation of a contracted gallbladder on repeated imaging studies despite the actual absence of the gallbladder, with the diagnosis ultimately established intraoperatively. Previously published literature has highlighted that the US is particularly misleading in differentiating GA from a severely fibrotic or atrophic gallbladder, especially in elderly patients and those with metabolic comorbidities, frequently resulting in an erroneous diagnosis of chronic cholecystitis and inappropriate surgical planning. The same study also describes intraoperative scenarios in which the gallbladder was unexpectedly absent, with the diagnosis of GA being established only during surgical exploration, thereby exposing surgeons to unanticipated technical difficulty and an increased risk of biliary injury [6].

Important differential diagnoses of gallbladder agenesis include a contracted gallbladder, scleroatrophic gallbladder, ectopic gallbladder, and intrahepatic gallbladder, all of which may present as non-visualization of the gallbladder on routine imaging studies.

MRCP is regarded as a highly sensitive modality for biliary evaluation and is frequently advocated when the gallbladder is not visualized on US. Although MRCP is considered the preferred non-invasive modality for evaluating the biliary tree, it has limitations in diagnosing gallbladder agenesis. Severe gallbladder contraction, fibrosis, poor visualization of the cystic duct, motion artifacts, and adjacent bowel loops may lead to misinterpretation of gallbladder absence as a contracted or shrunken gallbladder. Consequently, even MRCP may fail to establish a definitive preoperative diagnosis of gallbladder agenesis, as observed in the present case. Supporting literature indicates that high-resolution MRCP, particularly 3D sequences, may demonstrate absence of the gallbladder fossa or cystic duct, allowing preoperative diagnosis in selected cases [7]. The final diagnosis was congenital gallbladder agenesis. The term "vanished gallbladder" is used only to describe persistent radiological non-visualization of the gallbladder, which contributed to the diagnostic dilemma. However, as seen in our case, Bianco et al. also reported contradictory evidence suggesting that MRCP often fails to differentiate GA from a severely atrophied or “vanished” gallbladder, resulting in persistent mislabeling as contraction, as also observed in this case [8].

The second key challenge was the management of choledocholithiasis. The principal management challenge in this case was choledocholithiasis. Endoscopic clearance was unsuccessful despite sphincterotomy and balloon extraction, likely due to the large stone size. Temporary biliary stenting followed by laparoscopic exploration of the CBD enabled definitive management, highlighting the importance of a multidisciplinary approach in complex biliary pathology. As seen in our case, Husain et al. also reported that nearly half of symptomatic GA patients develop CBD stones, and stones >15 mm are associated with reduced ERCP success rates [9]. Several reports by Peloponissios et al. and Fiaschetti et al. have similarly highlighted the diagnostic difficulty of distinguishing gallbladder agenesis from a contracted gallbladder on preoperative imaging [10,11]. Failed endoscopic clearance, stent placement, and subsequent laparoscopic CBD exploration represent a well-described management pathway. The successful surgical extraction in this case, despite unexpected GA, highlights the importance of surgical preparedness and multidisciplinary planning.

Overall, this case emphasizes the need for heightened suspicion of GA in patients with persistent gallbladder non-visualization, awareness of imaging limitations, and a cautious intraoperative strategy to prevent unnecessary dissection and biliary injury, ultimately improving patient outcomes.

## Conclusions

GA is an uncommon cause of biliary symptoms and is frequently misdiagnosed due to misleading imaging findings. Persistent non-visualization of the gallbladder on serial ultrasonography and MRCP should prompt suspicion of agenesis rather than a contracted gallbladder. Despite advanced imaging, diagnosis is often made intraoperatively, necessitating surgical vigilance to avoid biliary injury. Awareness of this entity and appropriate use of ERCP and laparoscopic CBD exploration for associated choledocholithiasis are crucial to prevent misdiagnosis, unnecessary surgical intervention, and improve outcomes.
